# Impact of Stromal Deposit Depth on Pneumatic Dissection During DALK for TGFBI Corneal Dystrophies

**DOI:** 10.3390/jcm15030917

**Published:** 2026-01-23

**Authors:** Luca Lucchino, Giacomo Visioli, Giulio Pocobelli, Fabio Scarinci, Rossella Anna Maria Colabelli Gisoldi, Chiara Komaiha, Giacinta Buffon, Marco Marenco, Alessandro Lambiase, Augusto Pocobelli

**Affiliations:** 1 Department of Sense Organs, Sapienza University of Rome, 00185 Rome, Italy; luca.lucchino@uniroma1.it (L.L.);; 2 Vitreoretinal Service, Southend University Hospital NHS Foundation Trust, Prittlewell Chase, Westcliff-on-Sea SS0 0RY, UK; 3 UOC Oftalmologia-Banca Degli Occhi, San Giovanni Addolorata Hospital, 00184 Rome, Italy

**Keywords:** corneal stromal dystrophies, deep anterior lamellar keratoplasty, anterior segment optical coherence tomography, pneumatic dissection, big bubble

## Abstract

**Objectives**: To evaluate whether preoperative anterior segment optical coherence tomography (AS-OCT) parameters differ according to Big Bubble (BB) formation during deep anterior lamellar keratoplasty (DALK) in patients with TGFBI-related corneal stromal dystrophies (CSD). **Methods**: This retrospective cohort study included 17 eyes from 12 patients undergoing DALK with an attempted BB technique. Stromal deposit depth was assessed by AS-OCT using both a categorical depth-based classification (anterior, mid-, and posterior stroma) and continuous measurements of stromal involvement (µm). The ratio between stromal involvement and the thinnest corneal point was calculated. Intraoperative data included BB success, BB type, and complications. Inter-eye correlation was accounted for in comparisons of continuous variables using linear mixed-effects models. **Results**: BB formation was achieved in 11 of 17 eyes (64.7%), with type 1 BB observed in all successful cases. BB success was observed in all eyes with anterior or mid-stromal involvement and in 33.3% of eyes with posterior stromal involvement. Greater stromal deposit depth and a higher stromal-depth-to-thinnest-point ratio were observed in eyes in which BB formation failed (*p* < 0.01). No intraoperative perforations or conversions to penetrating keratoplasty occurred. Inter-observer agreement for AS-OCT measurements was high. **Conclusions**: BB failure was more frequent in eyes with greater absolute and relative stromal deposit depth, as assessed by preoperative AS-OCT during DALK in TGFBI-related CSD. These AS-OCT-derived parameters may support surgical planning and improve patient selection for BB DALK in this clinical setting.

## 1. Introduction

Corneal stromal dystrophies (CSD) comprise a group of bilateral, non-inflammatory, inherited disorders characterized by abnormal stromal deposits and a typically symmetric, slowly progressive course. They are classified according to clinical phenotype and underlying genetic mutations [1]. Mutations in the transforming growth factor beta–induced (TGFBI) gene account for several of the most common subtypes. Among these, Reis–Bücklers corneal dystrophy (RBCD), Thiel–Behnke corneal dystrophy (TBCD), lattice corneal dystrophy (LCD), and granular corneal dystrophies (GCD) are characterized by progressive stromal protein deposition, leading to recurrent corneal erosions, opacities, and visual impairment [1,2].

Management strategies for TGFBI dystrophies depend on disease severity. Recurrent erosions are generally managed with conservative measures, such as topical lubricants. For visually significant opacities, treatment options vary according to the depth of stromal involvement. Superficial opacities are typically treated with phototherapeutic keratectomy (PTK) or superficial anterior lamellar keratoplasty (SALK) [3,4]. Deep and diffuse stromal disease is best managed with deep anterior lamellar keratoplasty (DALK), which is preferred over penetrating keratoplasty (PK) in the absence of endothelial involvement [5]. DALK offers significant advantages over PK. The primary benefit of DALK is that it prevents endothelial rejection. In addition, it is associated with better preservation of endothelial cell density and a lower risk of postoperative ocular hypertension, suggesting a potential for longer graft survival [6,7]. Moreover, DALK enhances surgical safety by avoiding open-sky surgery and reducing susceptibility to wound dehiscence [8]. In addition, it requires less stringent criteria for donor corneal tissue selection, which may facilitate graft availability in clinical practice [9].

However, DALK is technically more demanding than PK, with a longer operative time and a steeper learning curve [10]. Its success relies on achieving a uniform and sufficiently deep stromal bed, which requires greater surgical expertise and may influence postoperative visual outcomes [11].

The Big Bubble (BB) technique, used in DALK, consists of a forced intrastromal air injection to achieve pneumatic dissection and expose the Descemet membrane (DM) [12]. Three BB types exist: type 1, formed between deep stroma and the pre-Descemet layer (PDL); type 2, formed between the PDL and DM; and a mixed type. The anatomical plane of separation has important surgical implications: type 1 BBs, the most common type, benefit from the presence of the PDL, which provides greater intraoperative stability, whereas type 2 BBs develop on the bare DM, making them extremely fragile and prone to rupture; they are an independent risk factor for conversion to PK [12,13].

While BB formation has been extensively studied in keratoconus and corneal scars, evidence on stromal dystrophies remain limited [14,15,16,17,18]. Recurrence of TGFBI dystrophies after DALK has been linked to the amount of residual stromal tissue [1,2,19]. Because BB DALK provides a thinner and more uniform stromal bed than manual dissection, identifying preoperative predictors of BB formation is especially relevant in CSD [20].

This study aims to evaluate whether specific topographic or anterior segment optical coherence tomography (AS-OCT) parameters are associated with the success of BB formation in patients with TGFBI corneal dystrophies.

## 2. Methods

This retrospective cohort study was approved by the Central Ethics Committee of Lazio, Italy (protocol number 0022/2024; Ref. 7468), and was conducted in adherence to the Declaration of Helsinki. The records of all DALK procedures performed on patients with TGFBI corneal dystrophies at San Giovanni Addolorata Hospital, Rome, Italy, from January 2021 to November 2023 were reviewed.

The inclusion criteria were as follows: patients with TGFBI CSD, diagnosed based on patient history, family history, and slit-lamp examination, with stromal opacity affecting the visual axis [21,22], aged ≥18 years, and underwent an attempted BB during DALK surgery. The exclusion criteria included prior PK, DALK surgeries, DM lesions or corneal perforations, previous refractive surgery, CSD not related to TGFBI, stromal opacities resulting from other etiologies (e.g., ocular burns, infectious keratitis, or trauma), primary ectatic disorders, systemic autoimmune conditions, and unreliable preoperative data.

Prior to undergoing the surgical procedure, all patients were subjected to a comprehensive ocular evaluation, which included corneal topography and AS-OCT with MS-39 (CSO, Florence, Italy) [15].

Data collection process encompassed a range of demographic information and clinical measurements. These included age, sex, K-max, K-mean, and the thinnest point.

**Figure 1:**
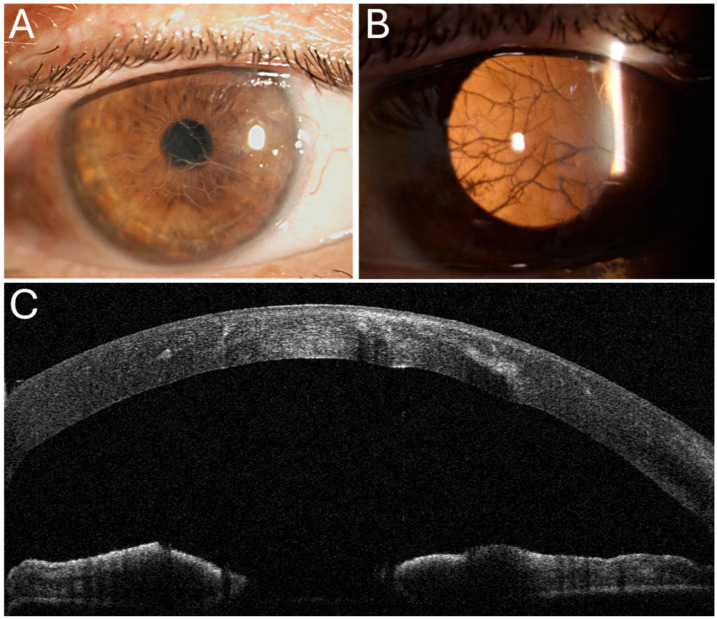
Slit lamp photographs and anterior segment optical coherence tomography (AS-OCT) of Lattice Corneal Dystrophy (LCD). (**A**) Slit-lamp examination under diffuse illumination shows stromal opacities with a branching, lattice-like pattern. (**B**) Retroillumination highlights the presence of the lattice-like deposits within the corneal stroma. (**C**) AS-OCT scan reveals hyperreflective intrastromal deposits predominantly involving the mid-stroma, with a posterior shadowing effect.

Before surgery the extent of stromal involvement was first assessed qualitatively on AS-OCT by identifying the deepest level reached by hyperreflective stromal deposits. For each eye, the stroma was divided into three equal-thickness zones (anterior stroma, mid-stroma, and posterior stroma), and eyes were classified according to the deepest affected zone, as previously described (Figure 1) [16,23].

To complement this categorical grading, stromal involvement was also quantified in micrometres (µm). Two independent investigators measured the maximum depth of hyperreflective stromal deposits using the manual calliper tool of the MS-39 device, and the average of their measurements was used for analysis. Finally, the ratio between stromal involvement depth and the thinnest point was calculated to account for interindividual differences in corneal thickness.

### 2.1. Surgical Technique

The BB technique was performed by a single surgeon (AP) as previously described [15]. Of note, trephination was performed using a pre-calibrated disposable suction trephine (Moria Surgical, Antony, France), with a diameter ranging from 8.00 to 8.50 mm. The intended depth of trephination was set at 90% of the lowest pachymetric value identified on the AS-OCT pachymetry map along the planned peripheral trephination zone. Prior to air injection, anterior keratectomy was performed in all subjects. A blunt Fogla spatula was then introduced to create an initial stromal track, followed by advancement of a 27-gauge Fogla cannula approximately 3 mm centripetally from the base of the trephination. Controlled air injection was performed until the edge of the trephination. A paracentesis was created to release aqueous humor and reduce intraocular pressure. Type 1 BBs were opened with a 23-gauge blade under viscoelastic protection, and the bubble roof was removed with blunt scissors. Type 2 BBs were collapsed at the periphery and followed by manual stromal dissection layer-by-layer; similarly, manual dissection was performed when BB formation was unsuccessful. Mixed-type bubbles were managed according to the predominant pattern. The donor cornea was then prepared using a suction punch of the same diameter as the receiving cornea. After staining with 0.05% trypan blue (RS-BLUE, ALCHIMIA, Padova, Italy), the donor DM–endothelium complex was removed, and the graft was secured with four interrupted 10-0 nylon sutures followed by a continuous running suture in sixteen passes.

The following intraoperative data were collected for each surgical procedure: the size and depth of trephination, the formation of a BB, the BB type (type 1, type 2, or mixed), micro/macroperforations of the DM, and any conversions to PK.

### 2.2. Statistical Analysis

The analysis was performed using STATA v. 18.0 (StataCorp LLC, College Station, TX, USA). Continuous variables were summarized using the median and interquartile range (IQR), while categorical variables were described as absolute numbers and percentages. Differences were assessed according to BB outcome. For continuous variables, comparisons between BB success and BB failure were performed using linear mixed-effects models with a random intercept at the patient level to account for inter-eye correlation in patients contributing both eyes [24]. Inter-observer reliability for stromal depth assessment was evaluated using the intraclass correlation coefficient (ICC). Discrepancies were resolved by a third investigator.

#### Baseline Characteristics of the Study Population

The study encompassed a total of 17 eyes from 12 patients (5 females) with a median age of 48 years (IQR 25). The cohort included ten patients with LCD, three with GCD, three with RBCD and one with TBCD. Patients were divided into three groups based on the depth of stromal involvement. Group 1 included four patients (23.5%: three RBCD, one TBCD) with opacities limited to the anterior stroma. All patients in this group had previously undergone PTK; however, the depth of recurrence exceeded the safe ablation range, and they were not considered suitable for repeat PTK. Group 2 comprised four patients (23.5%: three LCD, one GCD) with deposits in the intermediate stroma. Group 3 consisted of nine patients (52.94%: seven LCD, two GCD) with hyperreflective material extending to the deep stromal layers.

## 3. Results

Pneumatic dissection was achieved in 11 eyes (64.7%) with type 1 BB observed in all successful cases. Bubble formation was successful in 100% of cases in Groups 1 and 2, whereas in Group 3, it was achieved in only 33.3% of cases. Manual dissection was performed in all six cases (35.29%) where BB formation failed, with no perforations or need for PK conversion.

The preoperative characteristics of the included eyes, divided based on BB formation outcome, are presented in Table 1. Baseline parameters including dystrophy subtype distribution, trephination size, trephination depth, and corneal curvature indices (Kmean and Kmax) showed no appreciable differences between eyes with BB formation and those with BB failure. The thinnest corneal point was lower in eyes with BB failure compared with eyes with BB formation (median 447.5 µm vs. 540 µm). However, when accounting for inter-eye correlation using a linear mixed-effects model, the estimated difference was associated with substantial uncertainty (β = +58.4 µm for BB success; 95% CI −9.2 to +126.0).

In contrast, AS-OCT-derived stromal involvement depth was greater in eyes with BB failure (median 402.6 µm [IQR 74.3]) than in eyes with BB formation (median 274.5 µm [IQR 243.4]). In the mixed-effects model, the estimated difference for BB success was −133.3 µm (95% CI −233.7 to −32.9). The ratio between stromal involvement depth and the thinnest corneal point was higher in eyes with BB failure (median 0.86 [IQR 0.22]) compared with eyes with BB formation (median 0.51 [IQR 0.50]). The corresponding mixed-effects estimate for BB success was −0.324 (95% CI −0.547 to −0.100).

To date, no cases of clinically relevant recurrence have been observed. Inter-observer agreement for AS-OCT stromal depth assessment was high, with an intraclass correlation coefficient of 96.33%.

## 4. Discussion

In this series of patients with TGFBI-related corneal stromal dystrophies, AS-OCT-derived stromal depth was the parameter that most clearly differed between eyes with and without BB formation during DALK, with BB failure more frequently observed in cases with deeper stromal involvement. Overall, BB formation was achieved in 11 of 17 eyes (64.7%), a rate consistent with previously reported success rates ranging from 57.1% to 81% [17,18,25,26].

Previous studies assessed heterogeneous CSD cases without a systematic evaluation of stromal involvement, introducing a potential source of bias. Moreover, variability in surgeon expertise represents an additional factor that can influence surgical outcomes. To minimize these variables, we focused solely on TGFBI CSD, with all procedures performed by a single experienced surgeon and stromal depth evaluated via AS-OCT.

Li et al. and Huang et al. reported BB success rates of 76.6% (49/64) and 71.4% (20/28), respectively, across CSD subtypes including macular, granular, and lattice dystrophies. However, neither study evaluated stromal involvement [17,18]. Similarly, Bhatt et al. reported a 57.1% rate (4/7, LCD) without assessing stromal characteristics, and Unal et al., who used the largest available series (69 eyes, 81%), did not stratify outcomes by dystrophy subtype or consider stromal depth [25,26].

BB formation in CSD remains poorly studied compared to keratoconus, where larger trephination sizes and greater depth (90% partial thickness) have been associated with higher success rates [12,27,28]. Topographic and tomographic parameters have also been explored as predictive factors, though with conflicting results [14,15,29]. In this study, topographic parameters showed no significant differences between successful and failed BB cases. Nonetheless, measurement accuracy in CSDs may be affected by corneal opacity and surface irregularities, which can generate artifacts [30].

Recent studies have investigated the relationship between AS-OCT findings and BB formation in DALK, and the present study on CSDs further supports the association between stromal deposit depth and BB formation success [14,15,16,23]. Interestingly, the ratio between stromal involvement depth and the thinnest corneal point was higher in eyes in which BB formation failed. This observation could indicate that, in addition to absolute stromal depth, the relative extent of stromal involvement with respect to overall corneal thickness differs between BB success and failure. The present findings align with those of Borderie et al., who reported a 78% BB failure rate in cases with posterior corneal scars on AS-OCT [16]. However, their study included various corneal pathologies, with stromal dystrophies accounting for only 6 of 77 cases. A similar association has been reported in keratoconus patients, where higher AS-OCT stages and posterior stromal involvement have been associated with reduced BB formation rates or less favorable bubble characteristics, including an increased incidence of type 2 BBs [14,15].

In line with this, a previous study conducted in a homogeneous cohort of 33 patients with post-infective stromal scars highlighted a decline in BB success with increasing scarring depth. AS-OCT grading revealed success rates of 66.6% in the anterior stroma, 53.8% in the mid-stroma, and 14.3% in the posterior stroma [23]. The present study further refines the concept of stromal opacity depth by suggesting that BB outcomes are better captured using continuous AS-OCT-derived measurements, which complement depth-based staging by accounting for variability that may not be fully reflected by categorical classification. Moreover, the stromal involvement–thinnest point ratio represents a proportional measure that may help contextualize deposit depth in relation to overall corneal thickness, complementing absolute depth assessment.

TGFBI (also termed Keratoepithelin, TGFBIp, p68 β-ig-h3 or RGD-CAP) is an extracellular matrix protein predominantly produced by stromal keratocytes and corneal epithelial cells. It plays a critical role in maintaining the integrity of the corneal stroma, mediating adhesion between stromal collagen and the Descemet membrane [2]. Additionally, TGFBI has been shown to act as a growth factor, influencing several processes such as cell migration, differentiation, wound healing, and angiogenesis [2]. Mutations in *TGFBI* gene (5q31) cause protein misfolding, leading to insoluble extracellular deposits whose depth and distribution vary by dystrophy subtype [31]. Electron microscopy studies have shown that posterior stromal deposits disrupt collagen lamellae alignment and alter the extracellular matrix [32]. In this context, greater depth and overall stromal involvement of stromal deposits may interfere with microbubble formation, ultimately reducing the likelihood of successful pneumatic dissection.

In addition to the depth and extent of stromal involvement, the biochemical nature of TGFBI-related deposits, which varies according to the underlying genetic mutation, may also influence the success of pneumatic dissection. Among the most well-characterized genotype–phenotype associations, variants at Arg124 are typically associated with amyloid deposits, as observed in LCD, whereas mutations at Arg555 are linked to non-amyloid hyaline deposits, such as in GCD and RBD [33]. Hyaline deposits consist of truncated TGFBI protein fragments, whereas amyloid deposits are composed of full-length TGFBI protein and exhibit characteristic histopathological features, including Congo red positivity and birefringence under polarized light. These differences in deposit composition are accompanied by variability in stromal localization and spatial distribution across dystrophy subtypes [31]. Interestingly, alterations in stromal protein pattern have been reported to modify corneal biomechanical properties, with increased stromal stiffness and reduced corneal deformation described in LCD compared with healthy corneas, although the current evidence remains limited [34]. Such biomechanical changes may interfere with microbubble coalescence and expansion, thereby contributing to pneumatic dissection failure. In the present study, to explore whether deposit composition influenced BB formation, LCD was analyzed separately from other TGFBI stromal dystrophies. No significant differences in BB success were observed between these groups; however, this finding should be interpreted cautiously considering the limited sample size. Larger studies are warranted to determine whether, alongside stromal depth and extent, genotype-related differences may further inform surgical planning in TGFBI corneal dystrophies.

TGFBI stromal dystrophy recurrence has been documented in both DALK and PKP and correlates with follow-up duration [19,35,36]. Manual dissection techniques have been linked to earlier recurrence [22,37], whereas descemetic DALK may lower this risk by reducing residual host stroma [21,25]. Indeed, recurrence is believed to arise from repopulation of the graft by host keratocytes, which can resume producing mutant TGFBI protein; therefore, the amount of residual stromal tissue may play a key role in determining recurrence risk [37,38].

Chen et al. reported a higher recurrence rate after DALK (49.5% at 5 years) compared to PKP (40% at 10 years). However, the higher recurrence rate may reflect the use of manual dissection techniques in their study [37]. The only RCT comparing BB DALK and PK in macular dystrophy found comparable recurrence rates after 30 months (5.7% for DALK vs. 4.8% for PKP) [21].

Overall, recurrence rates of CSD after DALK vary widely, likely due to differences in surgical techniques, genetic variability, and follow-up duration. Additionally, the lack of distinction between simple and clinically significant recurrences may contribute to discrepancies across studies [19]. In our cohort, no significant recurrence has been observed to date; however, the short follow-up period precludes definitive conclusions. Notably, in cases of disease recurrence, repeat DALK offers a superior safety profile in comparison to repeat PKP, where immune-mediated endothelial rejection remains a major concern [38]. Based on previous studies, DALK should be prioritized over PKP whenever feasible in the surgical management of CSD, given its advantages in long-term graft survival and the potential for safer repeat procedures [21,25,35].

This study has several limitations. First, the sample size is relatively small; although this reflects the rarity of TGFBI stromal dystrophies and the strict inclusion criteria, it may limit the generalizability of the findings, and inferential statistical results should therefore be interpreted with caution. Second, the retrospective design may introduce selection bias. Moreover, the follow-up duration remains limited and is insufficient to assess long-term recurrence rates, which were beyond the primary scope of this study. Finally, although continuous AS-OCT-derived measurements were used to refine the assessment of stromal involvement, these parameters may not fully capture the spatial complexity and heterogeneity of stromal deposits. Future studies incorporating *en face* or volumetric imaging approaches may allow a more comprehensive characterization of stromal pathology [39,40].

In conclusion, this study shows that BB failure was more frequent in eyes with greater absolute and relative stromal deposit depth, as assessed by AS-OCT. These parameters may support surgical planning and patient selection for BB DALK in TGFBI-related CSD. Further studies with larger patient cohorts and extended follow-up periods, stratified by AS-OCT measurements of residual stromal thickness, are warranted to validate these findings and refine our understanding of surgical outcomes in TGFBI-related CSD.

## Figures and Tables

**Table 1:** Preoperative characteristics of the included eyes grouped by big bubble (BB) formation and BB failure during deep anterior lamellar keratoplasty (DALK). IQR, Interquartile range. Continuous variables are reported as median (IQR). *p*-values for continuous variables were adjusted for inter-eye correlation using mixed-effects models.

Parameter	Total (n. 17)	BB Formation (n. 11)	BB Failure (n. 6)	*p*-Value
Lattice Corneal Dystrophy, n (%)	10 (58.8%)	5 (45.5%)	5 (83.3%)	0.304
Trephination size (mm), median [IQR]	8.25 [0.25]	8.5 [0.25]	8.25 [0.13]	0.173
Trephination depth (µm) median [IQR]	540 [50]	500 [50]	450 [75]	0.193
Kmean (D), median [IQR]	43.6 [2.5]	43.4 [1.9]	44.7 [6.8]	0.199
Kmax (D), median [IQR]	46.5 [9.0]	45.8 [8.2]	48.9 [11.9]	0.353
Thinnest point (µm), median [IQR]	480 [105.5]	540 [131]	447.5 [86.25]	0.090
Stromal involvement depth (µm), median [IQR]	334 [194.6]	274.5 [243.4]	402.6 [74.3]	0.009
Ratio between stromal involvement and thinnest point	0.72 [0.35]	0.51 [0.50]	0.86 [0.22]	0.005

## Data Availability

The data that support the findings of this study are available from the corresponding author upon reasonable request.
